# Overcoming Anatomical Challenges in Difficult Cholecystectomies: A Narrative Review on the Impact of ICG in Patients with Obesity

**DOI:** 10.3390/life16050728

**Published:** 2026-04-25

**Authors:** Marcello Agosta, Giorgio Melita, Maria Sofia, Chiara Mazzone, Gloria Faletra, Gaetano La Greca, Saverio Latteri

**Affiliations:** 1 Department of General Surgery, Cannizzaro Hospital, 95126 Catania, Italy; marcello.agosta@studium.unict.it (M.A.); mariasofia2002@libero.it (M.S.); chiara.mazzone@phd.unict.it (C.M.); gloria.faletra@gmail.com (G.F.); glagreca@unict.it (G.L.G.); saverio.latteri@unict.it (S.L.); 2 Department of General Surgery and Medical-Surgical Specialties, University of Catania, 95123 Catania, Italy; 3 Department of Medical, Surgical Sciences and Advanced Technologies “G.F. Ingrassia”, University of Catania, 95123 Catania, Italy

**Keywords:** laparoscopic cholecystectomy, indocyanine green, near-infrared fluorescence cholangiography (NIRF-C), obesity, critical view of safety

## Abstract

Laparoscopic cholecystectomy is now established as the worldwide gold standard for the treatment of benign gallbladder disease. Despite technical advancements, bile duct injury (BDI) remains a major concern, especially in patients with obesity. It is well known that in patients with a Body Mass Index (BMI) ≥ 30 kg/m^2^, identification of Calot’s triangle and the achievement of the Critical View of Safety (CVS) during laparoscopic cholecystectomy (LC) are made more challenging due to excessive visceral adiposity and concomitant hepatic steatosis reducing the workspace. Near-Infrared Fluorescence Cholangiography (NIRF-C) with Indocyanine Green (ICG) has emerged as an innovative, safe and effective technique to visualize the biliary anatomy and minimize the risk of iatrogenic BDI. However, its specific benefit in patients with obesity remains under-discussed compared to the general population. The main aim of this narrative review is to evaluate whether the intraoperative use of ICG in patients with obesity may reduce operative times and the risk of BDI. A focused review of the literature is performed on articles from 2010 to 2025 published on PubMed, Scopus and Web of Science. The application of ICG fluorescence in LC for patients with obesity represents a tangible clinical advantage, not only for anatomical identification and significant improvement of procedural efficiency, but also for the reduction in operative time.

## 1. Introduction

### 1.1. Background and Epidemiology

Obesity is defined by the World Health Organization (WHO) as excess fat accumulation that increases risk for adverse health outcomes [1], including chronic diseases such as cardiovascular diseases, type 2 diabetes, and gastrointestinal disorders [1,2]. Today, obesity is considered one of the major public health challenges worldwide and is currently rising at an alarming rate [3,4]. A newly published study reveals that in 2022, the number of people with obesity amounted to 880 million, with an 18.5% prevalence in the female population and 14% prevalence in the male population. It also indicates how the prevalence of obesity among children and adolescents (5–19 years old) has quadrupled [4]. Clinical classification is based on the BMI (Body Mass Index), calculated by dividing the body weight in kilograms by height in meters squared, where a BMI ≥ 30 kg/m^2^ identifies class I obesity and a BMI ≥ 35 kg/m^2^ indicates class II or severe obesity.

### 1.2. Obesity, Rapid Weight Loss and Gallstone Disease

Among the correlated gastrointestinal pathologies, cholelithiasis represents a predominant healthcare burden. A recent systematic review and meta-analysis from 2024 indicates that 6% of the population has gallstones and that the incidence of this condition may be increasing [5]. It is well known that both excess of body fat and rapid weight loss after bariatric surgery are related to gallstone formation [6,7,8]. On the one hand, obesity favors the formation of cholesterol gallstones through metabolic alterations, hepatic secretion of supersaturated bile, dyslipidemia and decreased secretion of bile acids [6]. On the other hand, the increasing incidence of cholelithiasis following bariatric surgery may be explained by rapid fat mobilization, followed by the rise in serum cholesterol and triglyceride levels associated with gallbladder contractile dysfunction caused by decreased cholecystokinin levels [9].

### 1.3. Laparoscopic Cholecystectomy: Challenges in Obesity

**Figure 1:**
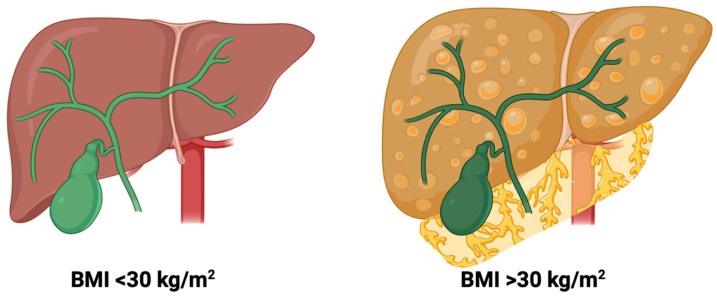
Anatomical challenges in patients with obesity during laparoscopic cholecystectomy. Created in BioRender. Augello, E. (2026) https://BioRender.com/v0div5t (accessed on 19 April 2026).

Laparoscopic cholecystectomy is universally considered the gold standard for treating symptomatic cholelithiasis, having supplanted open surgery [10]. However, laparoscopic techniques have led to an increase in the incidence of other complications, especially BDI (bile duct injury), a rare yet potentially severe complication associated with significant morbidity, a long-term decrease in quality of life, and potential mortality [11,12,13]. In response to increasing complication rates, the Critical View of Safety (CVS) was introduced as a preventative measure to decrease the risk of injury to extrahepatic bile ducts [14]. BDI is a serious issue among patients with obesity (BMI ≥ 30 kg/m^2^) and especially in those with severe obesity (BMI ≥ 35 kg/m^2^), in whom excessive visceral adiposity and a steatotic liver are factors that hinder the safe identification of Calot’s triangle and the achievement of the CVS, thereby not only increasing the risk of iatrogenic BDI, but also significantly prolonging operative times [15,16]. A careful dissection of the fibro-fatty tissue within this triangle is required to achieve the CVS [14]. Figure 1 illustrates how excessive visceral adiposity and hepatic steatosis in patients with obesity (BMI > 30 kg/m^2^) obscure the hepatobiliary triangle and hinder the identification of biliary structures required to achieve the CVS.

### 1.4. Role of Indocyanine Green

To mitigate these risks, Near-Infrared Fluorescence Cholangiography (NIRF-C) with Indocyanine Green (ICG) has emerged as a safe, noninvasive, real-time, radiation-free imaging method that uses fluorescence for visualizing biliary anatomy during surgery [17,18]. ICG is a water-soluble tricarbocyanine with a molecular weight of 774.96 g/mol, absorbing excitation light at 800–835 nm while emitting fluorescent light at 740–793 nm. ICG binds with circulating albumins and lipoproteins and is excreted into the bile almost unaltered following hepatic extraction. It is mostly administered intravenously within 1 h before imaging; an alternative in biliary surgery is its direct injection into the gallbladder [19]. The use of ICG facilitates the identification of the cystic duct and the common bile duct even in the hostile anatomical conditions typical of patients with obesity, offering a potential advantage in terms of reducing operative times and preventing major complications.

### 1.5. Research Gap and Aims

Despite the widespread use and proven utility of NIRF-C, the literature still lacks studies that allow us to fully overcome certain critical issues regarding the actual role of this innovative technique in patients with obesity. First, there is a limited availability of studies evaluating a BMI ≥ 30 kg/m^2^ as an independent parameter to establish the benefit of ICG specifically in reducing the dissection time of Calot’s triangle. Indeed, most of the studies tend to group patients with obesity within the common category of “difficult cholecystectomies” (along with acute cholecystitis, cirrhosis and previous surgery). Second, there is an unresolved controversy regarding its procedural advantage in patients with a BMI ≥ 35 kg/m^2^. According to Serban et al. and Pesce A et al., the thickness of visceral fat may exceed the penetration capacity of near-infrared (NIR) light, paradoxically reducing the effectiveness of the method [20,21].

Considering the points discussed above, the primary objective of the present review is to evaluate whether, based on the current evidence in the literature, the routine use of ICG in patients with obesity (BMI ≥ 30 kg/m^2^) yields a statistically significant reduction in total operative time and, specifically, the time required to achieve the CVS compared to standard white-light laparoscopy. The second objective is to define the limitations of this technology in relation to BMI, determining whether there is a specific Body Mass Index cut-off beyond which the penetration of the NIR signal becomes ineffective.

## 2. Materials and Methods

A rigorous literature search was conducted across the PubMed, Scopus, and Web of Science databases, covering the period from 2010 to 2025. The search strategy utilized specific keywords, including “Near-Infrared Fluorescence Cholangiography” (NIRF-C), “Indocyanine Green” (ICG), “laparoscopic cholecystectomy”, and “Critical View of Safety”. A total of 233 articles were identified across the various databases; of these, 54 articles were selected for further eligibility screening, and ultimately, 23 articles were included in the final review. Clinically, inclusion was restricted exclusively to elective laparoscopic cholecystectomies performed for benign biliary disease. Methodologically, we prioritized high-level evidence designs, specifically meta-analyses, randomized controlled trials (RCTs), and cohort studies. It must be acknowledged, however, that primary data specifically dedicated to the population with obesity remains relatively limited, with few studies evaluating a BMI of 30 kg/m^2^ as an independent parameter. Consequently, several foundational studies involving broader surgical populations were included to definitively validate the general efficacy and safety profile of the NIRF-C technique. These selected studies provided BMI reporting or focused on the anatomical challenges associated with visceral adiposity. This approach allows for a rigorous evaluation of whether the established benefits of ICG—such as real-time anatomical visualization and reduced operative duration—are consistently maintained in the presence of the unique technical barriers posed by a high BMI.

## 3. Discussion

### 3.1. The Critical View of Safety

Laparoscopic cholecystectomy represents the current standard of care for patients with uncomplicated cholelithiasis and other benign gallbladder diseases. The development of laparoscopy radically transformed the abdominal surgical approach, guaranteeing accelerated postoperative recovery, reduced pain, and superior cosmetic results compared to open surgery. However, despite the improvement in surgical techniques and a globally consolidated learning curve, the incidence of iatrogenic BDIs remains a persistent critical issue, with an incidence rate ranging between 0.3% and 0.7% [21]. This complication results in devastating morbidity for the patient and high healthcare costs and its primary cause—identified in 71–97% of cases—lies in the surgeon’s misinterpretation of biliary anatomy [21]. The risk of BDIs is amplified by factors distorting or obscuring the anatomy of the hepatocystic triangle (or Calot’s triangle). This area contains critical structures such as the cystic artery and cystic duct that must be identified and carefully isolated prior to dissection of the overlying fibro-adipose tissue. It is reasonable to assume that among these risk factors, obesity emerges as one of the most complex and widespread challenges in modern surgery, as the accumulation of visceral adipose tissue overlies the structures of the hepatic hilum, reducing maneuverable space and making dissection of Calot’s triangle more challenging. In addition, steatosis hepatomegaly—frequent in these patients—limits retraction and exposure of the surgical field. In this context, achieving the CVS becomes technically demanding and necessitates prolonged dissection times (99.0 min for a BMI < 30 vs. 104.9 min for a BMI > 30) [21]. The standard classification of obesity is based on BMI; however, this measure does not distinguish between the type of adipose tissue or location of fat within the intra-abdominal cavity [22,23]. In order to assess the effects of intra-abdominal adipose tissue, S. L. Vlek et al. suggested that the patients should be selected on the basis of fat percentage assessed by either CT or MRI instead of BMI [18]. To overcome these challenges, NIRF-C using ICG has emerged as one of the most promising innovations, allowing for real-time visualization of the extrahepatic biliary tree through overlying tissues and successfully reducing operative times (42.6 min vs. 48.3 min) [24].

### 3.2. Indocyanine Green Fluorescence: Mechanism, Dosage, and Administration Protocols

ICG is a tricarbocyanine sterile dye that binds almost exclusively to circulating albumin and lipoproteins and is excreted into the bile with minimal metabolic alteration. It has an excellent safety profile for human use; however, according to the pharmacopeia, ICG contains iodide impurities; therefore, allergy to iodine is a critical factor [25]. The molecule of ICG has an emission with a spectrum peak at 810–830 nm and when illuminated by near-infrared light (750–810 nm), the protein-bound ICG in the bile duct fluoresces, penetrating overlying connective and adipose tissue. The optimal dosing and timing of administration for patients with obesity are still debated among the scientific community. The most used method of administration of ICG is intravenously. Only one study evaluated the efficiency of NIRF-C when ICG was administered directly into the gallbladder [19]. Dip et al. used a weight-based protocol (0.05 mg/kg) with intravenous administration 1 h before surgery to specifically compare accuracy between patients with a BMI > 30 and patients with a BMI < 30 [26]. In a randomized trial, Dip et al. administered the same dose but 45 min before surgery [27]. In contrast, Broderick et al. adopted a standard protocol consisting of a 7.5 mg dose administered at least 45 min prior to surgical intervention [28]. Results of an international Delphi survey indicate that a weight-based protocol (>0.05 mg/kg) represents the most appropriate approach [29]; Jacqueline van den Bos et al. demonstrated that when using a 2.5 mg bolus, the cystic duct was visualized in an average percentage of 94% patients, while when using 0.05 mg/kg, the average percentage of visualization of the cystic duct was 98% [30]. Even though, in a more recent study, Broderick et al. showed that implementation of a microdose ICG protocol of 0.5 mg provides non-inferior biliary visualization compared to the historical standard protocol, with equivalent operative time and perioperative outcomes, the analysis is limited by the lack of adjustment for factors such as obesity [31]. The administration protocols for Indocyanine Green (ICG) are summarized in Table 1.

### 3.3. Biliary Tract Visualization in Patients with Obesity: Efficacy and Limitations

A major concern regarding the effective advantage of NIRF-C with ICG is the limited ability of near-infrared light to penetrate the thick fatty tissue encasing the structures of the biliary tree. Several studies suggest that the penetration ability of near-infrared light is limited to 5–10 mm [20,21,26,32]. In patients with severe obesity (BMI > 35), the volume of visceral fat surrounding the hepatobiliary triangle frequently exceeds the penetration threshold of near-infrared light. Therefore, NIRF-C should be utilized as a dynamic, real-time navigation tool rather than a static predissection map. Its maximal clinical utility is achieved following the initial dissection of Calot’s triangle; once this barrier is thinned, the reduction in distance allows the NIR signal to clearly delineate the underlying biliary structures, facilitating the safe completion of the CVS. To efficiently obtain fluorescence signals, it is important to set the tip of the 0° or 30° laparoscope vertically to Calot’s triangle to directly irradiate exciting light on the bile ducts [20]; in addition, ex vivo experiments have demonstrated that increasing the operating distance of the IGC solution from 5 to 14 cm leads to a reduction in fluorescence intensity by 5 to 30 times [30]. Beyond the limitations of BMI as a purely anthropometric measure, preoperative assessment of intra-abdominal fat distribution—rather than total fat percentage alone—may directly influence the technical execution of NIRF-C. In patients with significant visceral adiposity, standard port placement may require adjustment, often shifting more cranially or laterally, to facilitate a vertical and direct trajectory toward the hepatocystic triangle. Achieving this perpendicular alignment is critical to maximize the irradiation of exciting light on the biliary structures, especially given the limited penetration threshold of NIR rays. Furthermore, surgeons should prioritize maintaining the laparoscope at the minimum possible distance from the targeted tissue, as fluorescence intensity can decrease by up to 30 times when the operative distance increases. Despite this, several studies report that NIRF-C remains a more effective tool compared to standard white-light imaging or intraoperative cholangiography (IOC), specifically concerning the visualization of the cystic duct. Osayi et al. compared the use of NIRF-C with intraoperative cholangiography, demonstrating that it is less time-consuming (1.9 ± 1.7 min for NIRF-C vs. 11.8 ± 5.3 min for IOC) and that there is improved visualization of biliary structures with NIRF-C in patients with a BMI < 30 kg/m^2^ compared to those with a BMI > 30 kg/m^2^, though this was only significant for visualization of the CD-CHD junction. In all patients, the CD was visualized at a significantly higher rate with NIRF-C [33]. In a randomized trial, Dip et al. found that there was a mean 6% reduction in biliary visualization per unit BMI increase; however, visualization always remained better with NIRF-C versus white light. Predissection detection rates were significantly superior in the NIRF-C group for the cystic duct, common hepatic duct and common bile duct, compared to the white-light group [27]. Pesce et al. found a modestly improved rate for the identification of biliary structures during LC with NIRF-C patients with a BMI < 30 kg/m^2^ relative to those with a BMI > 30 kg/m^2^. Only a statistical difference for the visualization of the CD CHD junction emerged; however, according to the data available no statistically significant difference exists between patients with a BMI < 30 kg/m^2^ and patients with a BMI > 30 kg/m^2^, regarding improved visualization of the biliary structures [34]. However, a critical distinction emerges when stratifying by obesity class. While ICG provides a reliable predissection advantage in class I obesity (BMI 30–34.9 kg/m^2^), structural detection becomes significantly harder in class II and III obesity (BMI ≥ 35 kg/m^2^). In cases of severe obesity, where the visceral fat layer easily exceeds the aforementioned penetration threshold, NIRF-C can fail to recognize predissection structures entirely, thus mandating a more iterative, dissection-dependent approach to safely achieve the CVS [35]. The percentages of successful biliary structure visualization are reported in Table 2.

### 3.4. Reduction in Operative Time and Surgical Conversions

Beyond its primary role in anatomical identification, one of the most compelling clinical benefits of NIRF-C has proven to be its ability to reduce both operative times and conversion rate. Increased operative time has been associated with increased infectious complications and length of hospital stay at a rate of 6% per hour, substantiating how the decrease in procedural duration offers an effective benefit, especially in patients with obesity [36]. A meta-analysis by Dip et al. provides high-level evidence regarding the clinical outcomes of NIRF-C compared to standard white-light imaging [37]. The overall weighted BDI rate was 6 per 10,000 patients with NIRFC, compared to 25 per 10,000 without it; NIRF-C was also associated with a weighted conversion rate of 16 per 10,000, while the rate without NIRF-C was significantly higher at 271 per 10,000. Specifically, among patients undergoing laparoscopic cholecystectomy, BDI rates were 0 vs. 32 per 10,000 and conversion rates were 23 vs. 255 per 10,000 (ICG vs. non-ICG) However, authors report a potential source of bias due to the lack of adjustment for factors such as obesity. In a recent systematic review conducted by Manasseh et al. [38], ICG significantly reduced operative times in complex cases (including obesity), with an average reduction of approximately 20 min compared to white light (*p* < 0.0001). Similarly, Ravikumar et al. found no bile duct injuries or conversions to open surgery and a reduced operative time with ICG fluorescence cholangiography compared to white light only (mean: 42.6 ± 5.2 min vs. 48.3 ± 6.1 min; *p* = 0.002). Furthermore, the hospital stay was significantly shorter in the ICG group (median: 1 day vs. 1.3 days; *p* = 0.045) [24]. Broderick et al. specifically calculated operative times in patients with obesity, demonstrating how ICG reduced operative time by 26.47 min per case (*p* < 0.0001). For patients with a BMI > 30 kg/m^2^ the operative time was 75.57 vs. 104.9 min (*p* < 0.0001), and the reduction in the conversion rate to open surgery remained statistically significant on multivariable analysis (Odds Ratio 0.212, *p* = 0.001) [28]. Overall, in the largest meta-analysis by Lie et al. which included 3457 patients, it was reported that ICG significantly reduced the operative time compared to white light (81.24 min vs. 97.3 min, *p* = 0.007). This reduction corresponded to a Standardized Mean Difference (SMD) of −0.86 (95% CI −1.49 to −0.23; I2 = 97%). Furthermore, the technique demonstrated a significantly lower conversion rate with a Risk Ratio (RR) of 0.28 (95% CI 0.16–0.50; *p* < 0.0001; I2 = 0%) [39]. Conversely, Symeonidis et al., in an RCT of 160 patients, observed no significant difference in operative times between the ICG group and the WL group (46.5 ± 7.43 min vs. 47.1 ± 7.31 min, *p* = 0.858), suggesting that ICG may not provide a substantial time-saving benefit in routine cases [40]. Data regarding operative times and conversion rates are detailed in Table 3.

## 4. Conclusions

The integration of NIRF-C with ICG into laparoscopic cholecystectomy offers a promising approach to address the technical challenges inherent in patients with obesity. In this population, visceral adiposity and hepatic steatosis often obscure the hepatobiliary triangle, potentially limiting the effectiveness of standard white-light visualization. The current literature suggests that ICG fluorescence may serve as a helpful navigational tool, providing real-time visualization of biliary structures through overlying tissues. Some studies suggest a potential reduction in operative times—with reported savings of up to 29 min in obese cohorts—and a possible decrease in conversion rates to open surgery. However, these findings are not uniform across all trials, highlighting the impact of study heterogeneity and the need for further high-quality evidence. Significant technical considerations remain, such as the limited penetration of near-infrared light, which is generally restricted to 5–10 mm. Consequently, a clear clinical distinction must be made: while ICG provides a tangible navigational advantage in class I obesity (BMI 30–34.9 kg/m^2^), its efficacy in class II and III (BMI ≥ 35 kg/m^2^) is often hindered because fatty tissue thickness exceeds this penetration range, requiring a more iterative, dissection-dependent approach to safely achieve the CVS. Furthermore, while ICG fluorescence may facilitate the achievement of the CVS, its direct impact on reducing the incidence of BDI remains difficult to confirm statistically within the currently available studies. Optimal outcomes appear to be supported by weight-based or low-dose administration protocols (e.g., 0.05 mg/kg), which may improve ductal contrast while minimizing interference from the steatotic liver. In conclusion, while ICG fluorescence represents a valuable safety adjuvant in the surgical management of patients with obesity, it should be considered a supplementary tool rather than a definitive standard of care. Future research should prioritize prospective, BMI-stratified analyses to more accurately define its clinical utility and the specific BMI thresholds at which its benefit may be limited by tissue thickness.

## 5. Limitations

Several limitations must be acknowledged when interpreting the results of this narrative review. Although NIRF-C with ICG appears to be a valuable and cost-effective tool that reduces operative duration and conversion rates, its clinical application is limited by significant methodological heterogeneity regarding dosing and timing. The lack of specific **BMI-stratified data** in the literature hinders the ability to address technical constraints, particularly the 5–10 mm penetration limit of near-infrared light, which may be insufficient for the tissue thickness in the case of severe obesity. Regarding the cost–benefit analysis, current predictive models suggest that routine Near-Infrared Fluorescence NIRF-C is a dominant and cost-effective strategy. Evidence indicates it may reduce lifetime costs by significantly decreasing operative duration and the rate of conversion to open surgery [41]. However, these economic evaluations are often based on retrospective data and single-institution contract agreements, meaning that true costs to other hospitals may vary based on specific device and pharmaceutical contracts. Furthermore, there is still a lack of prospective studies that weigh these costs against long-term outcomes specifically within the population with high BMI. 

## Figures and Tables

**Table 1:** Indocyanine Green (ICG) administration protocols. Comparison of fixed doses, weight-based dosing, and timing.

Study	Administration Mode	Dosage	Timing (Pre-Incision)	Notes for Patients with Obesity
Dip et al. (2016) [26]	Weight-based	0.05 mg/kg	~60 min prior	Recommended dosage for obese patients to compensate for distribution volume.
Dip et al. (2019) [27]	Weight-based	0.05 mg/kg	45 min prior	Standard protocol used in the largest multicenter RCT.
Broderick et al. (2021) [28]	Standard Fixed Dose	7.5 mg (3 mL)	>45 min prior	Historical protocol effective in reducing conversions and time even for a BMI > 30
Dip et al. (2022) [29]	Expert Consensus	>0.05 mg/kg	At least 45 min prior	Experts agree on weight-based dosing for greater accuracy.
Jacqueline van den Bos et al. [30]	Weight-based	0.05 mg/kg	15 min to 2 h prior	Average percentage of visualization of the cystic duct was 98%.
Broderick et al. (2025) [31]	Microdose Protocol	0.5 mg	16.7 ± 5.6 min prior	It lacks adjustment for various factors such as obesity.

**Table 2:** Biliary structure visualization rates (%). Comparison between ICG and white light (WL) and the impact of obesity. CD: cystic duct; CHD: common hepatic duct; CBD: common bile duct.

Study	Population/Comparison	CD	CHD	CBD	Significance
Dip et al. (2016) [26]	Obese vs. non-obese	100% (obese) vs. 100% (non-obese)	60.5% (obese) vs. 81.8% (non-obese)	81.6% (obese) vs. 93.9% (non-obese)	CHD visualization significantly decreases in obese patients (*p* = 0.04).
Osayi et al. (2015) [33]	BMI > 30 vs. BMI < 30	92.3% (obese) vs. 97.7% (non-obese)	61.5% (obese) vs. 76.7% (non-obese)	71.8% (obese) vs. 81.4% (non-obese)	No significant difference. Successful visualization even with BMI of 63 kg/m^2^.
Dip et al. (2019) [27]	ICG vs. WL (Predissection)	66.6% (ICG) vs. 36.2% (WL)	28.9% (ICG) vs. 10.9% (WL)	49.4% (ICG) vs. 20.6% (WL)	ICG is significantly superior to WL (*p* < 0.001).
Pesce et al. (2015) [34]	Systematic Review (BMI > 35 kg/m^2^ vs. <35 kg/m^2^)	91% (>35 kg/m^2^) vs. 92.3% (<35kg/m^2^)	N/A	64% (>35 kg/m^2^) vs. 71.8% (<35 kg/m^2^)	Above BMI of 35, CBD visualization drops, but CD visualization remains high (>90%).
Piccolo et al. (2023) [35]	“Difficult” Group (BMI > 35 kg/m^2^, cirrhosis)	81.8% (Difficult)	45.5% (Difficult)	72.7% (Difficult)	High failure rates in BMI > 35 kg/m^2^ due to tissue thickness >5–10 mm.

**Table 3:** Impact on operative times and conversion rates. Focus on time reduction and risk in obese vs. non-obese patients.

Study	Population Analyzed	Operative Time (min): ICG vs. No-ICG	Time Reduction	Conversion Rate: ICG vs. No-ICG	Notes
Dip et al. (2021) [37]	Meta-analysis (Total)	-	-	0.16% vs. 2.71%	Massive reduction in open conversion risk with ICG. BDI rates dropped to 0 vs. 32 per 10,000.
Manasseh et al. (2024) [38]	Systematic review	21.3 min to 117 min with ICG vs. 46.1 min to 137 min with No-ICG	~20 min (mean)	Trend favoring ICG	Time savings are maximized in “difficult cases” (including obesity).
Ravikumar et al. (2025) [24]	RCT (BMI not specified)	42.6 (ICG) vs. 48.3 (No-ICG)	−5.7 min (*p* = 0.002)	0% vs. 0%	Significant reduction even in general randomized cases.
Broderick et al. (2021) [28]	Patients with obesity (BMI >30)	75.6 (ICG) vs. 104.9 (No-ICG)	−29.3 min (*p* < 0.0001)	1.57% vs. 7.42%	ICG drastically reduces times and conversions in obese patients. Multivariable OR for conversion: 0.212 (*p* = 0.001).
Lie et al. (2023) [39]	Meta-analysis (3457 patients)	81.24 (ICG) vs. 97.3 (No-ICG)	−16.05 min (SMD −0.86, 95% CI −1.49 to −0.23, *p* = 0.007)	ICG associated with lower conversion rate. RR 0.28 (95% CI 0.16–0.50, *p* < 0.0001, I2 = 0%)	Reduction of 72% in conversion risk. Tissue thickness in obesity limits light penetration to 5–10 mm.
Symeonidis et al. (2024) [40]	Randomized control trial (ICG vs. IOC)	46.5 (± 7.43) vs. 47.1 (± 7.31) min	−0.6 min (not significant) (*p* = 0.858)	0% vs. 3.75%	ICG-FC was significantly faster to perform (1.8 min vs. 5 min) than standard IOC.

## Data Availability

No new data were created or analyzed in this study.

## Abbreviations

The following abbreviations are used in this manuscript:

BDI	Bile Duct Injury
BMI	Body Mass Index
CVS	Critical View of Safety
LC	Laparoscopic Cholecystectomy
NIRF-C	Near-Infrared Fluorescence Cholangiography
ICG	Indocyanine Green
WHO	World Health Organization
CHD	Common Hepatic Duct
IOC	Intraoperative Cholangiography
